# Supplementary material for: Optimizing time and split application of nitrogen fertilizer to harness grain yield and quality of bread wheat (Triticum Aestivum L.) in northwestern Ethiopia
Source: PLoS One. 2022 Dec 15;17(12):e0279193. doi: 10.1371/journal.pone.0279193 (PMC10045545; doi:10.1371/journal.pone.0279193)
Supplement: Attachment — Submitted filename: Comments_Plos One_D-22-18317.docx [file pone.0279193.s002.docx]

**Abstract**

It’s missing full stop and the end of the first sentence.

Name the treatments

What is the difference between all urea at booting and all N at booting

it can concluded, correct sentence is “It can be concluded”.

The abstract is sufficiently clear and focused on the main results of the work

**Introduction**

“Despite its importance, the productivity of wheat in Ethiopia under rain fed season is low

particularly in Amhara region (2.79)” what is the unit of 2.79?

“Low soil fertility, improper fertilizer application, lack of appropriate seeding, feretilizer

rate; and planting methods and pests are among the major constraints limiting wheat production

in Ethiopia [12, 13].”

Write fertilizer rate instead of feretilizer rate, replace “;” by “,” and remove “and” before planting methods

“To reduce these

challenges increasing the N use efficiency through different strategies such as (i) use of

appropriate type of fertilizer and application time during the crop growing period, (ii) application

of optimum N fertilizer rate, (iii) appropriate method of application, (iv) use of appropriate

cultivar and (v) application at the appropriate climatic conditions [21].”

There is no verb in this sentence

“Thus, nitrogen scheduling plays a vital role in

the growth, production, and quality of wheat as well as in its use efficiency [20].”

Somes artciles focused on the effect of N splitting on wheat <https://doi.org/10.1016/j.eja.2004.12.001>;

<https://doi.org/10.1016/S0378-4290(03)00167-9>;

https://doi.org/ 10.1016/S0378-4290(03)00167-9

https://doi.org/ 10.3389/fpls.2016.00738

“Thus, the main objective of this research was to determine the

appropriate N fertilizer application time for maximum bread wheat yield production with

optimum protein contenet.” Typing error prtein contenet. Write protein content…

“…wheat growing stags with various ratios of N across…” write growing stages

**Materials and Methods**

**Description of Experimental Sites**

A field experiment was conducted in three wheat production potentail areas of…

Potential areas of…

The manuscript is full of typing errors

**“…** districts of Amhara Region, Ethiopia (Figure 1) …” Follow the journal's instructions for citing figures and tables in the manuscript.

“Generally, the rainfall in the study areas follows a dominantly unimodal distribution with the

main peak in June to September,” Does this mean June is rainy as July, August and September? Main peak should be the rainiest month

“Geographically, Adet is located at 11o16' N latitude and 37o29' E longitude with an altitude of 2240 meters above sea level (m.a.s.l.) It has a long year mean annual rainfall of 1211mm with a minimum and maximum temperature of 11.57°C and 26.89°C respectively [33]. Wonberema is located at 10°27' N latitude and 37°56' E, longitude with an altitude of 2600 m.a.s.l. It has a long year mean annual rainfall of 1211mm with a minimum and maximum temperature of 17°C and 25°C, respectively”

The mean annual rainfall mentioned here is less than the rainfall recorded during the growing season in each of the 3 areas (Figure 2). How do authors explain this? Are they mean annual rainfall calculated over a period?

“In Debre Elias and Wonberema, the rain falls increased from 2014 and 2015 by

71.43% and 14.29%, respectively, ...” write ...from 2014 to 2015 by 71.43% and 14.29%, respectively,

“At *Adet* the soil textural class is clay with the soil pH, OC (g kg-1), TN (mg kg-1), Ava.P (mg kg-1), CEC (meq100g-1) of 5.4, 10.7, 966, 27.69 and 31.2, respectively [35]. The soil pH , OC(%), TN (%), Ava.P (ppm) and Av.K (ppm) at *Wonberema* were 5.7, 3.1, 0.3, 369 and 127, respectively with sandy loam textural lass [36]. The soil pH, OM (%), TN (%), Ava.P (mg kg-1) and CEC (Cmole(+) kg-1) at *Debre Elias* were 5.12, 2.63, 0.14, 2.53 and 25.02, respectively”

Better to present this in a table and make comments.

Having 966 mgN/kg of soil at Adet, no need to conduct such study on N splitting there. The soil contents much N than plant required. Chek this result. Same remark for Ava.P at Wonderema (369mg/kg). Why authors present TN results in different unit (mg kg-1 and %) in the same study. Please harmonize it. ppm = mg kg-1

Morever, the soil characterics presented are different from location to location. For instance, at Wonberema, authors don’t present the soil texture and CEC value and at Debre no soil texture presented. Harmonize this since soil texture and CEC impact plant response

**Treatments, Experiment Design and Planting Proceduers**

1. ½ urea at 50% emergence (Zadoks Growth Stage 11-18) + ½ urea at tillering (Zadoks

Growth Stage 21-26)

2. ½ urea at tillering (Zadoks Growth Stage 21-26) + ½ urea at booting (Zadoks Growth

stage 41- 45)

8

3. 1/3rd urea at 50% emergence (Zadoks Growth Stage 11-18) + 1/3rd urea at tillering

(Zadoks Growth Stage 21-26) + 1/3rd urea at booting (Zadoks Growth stage 41- 45)

4. 2/3rd urea at tillering (Zadoks Growth Stage 21-26) + 1/3rd urea at booting (Zadoks

Growth stage 41-45)

5. All urea at tillering (Zadoks Growth Stage 21- 26)

6. All urea at booting (Zadoks Growth stage 41- 45)

7. All N at tillering (Zadoks Growth Stage 21- 26)

8. All N at booting (Zadoks Growth stage 41- 45)

9. ½ N at planting (Zadoks Growth Stage 00) + ½ N at tillering (Zadoks Growth Stage 21-

26)

10. ½ N 50% emergence + ½ N at tillering (Zadoks Growth Stage 21- 26)

11. 1/3rd N at 50% emergence (Zadoks Growth Stage 11-18) + 1/3rd N at tillering (Zadoks

Growth Stage 21-26) + 1/3rd N at booting (Zadoks Growth stage 41- 45)

12. ½ N at tillering (Zadoks Growth Stage 21-26) + ½ N at booting (Zadoks Growth stage

41- 45)

I suggest authors to present this in table to facilitate the reading to readers

For instance, a table like the following

| Treatment | Nitrogen application | | |
| --- | --- | --- | --- |
|  | 1^st^ application | 2^nd^ application | 3^rd^ application |
| 1 |  |  |  |
| 2 |  |  |  |

“Note that, from treatment 1 to 6, N was applied with Di-Ammonium Phosphate (DAP) at planting and from treatment 7 to 12 no N was applied at planting instead TSP (Tri- Supper Phosphate) was used.”

“Note that, from treatment 1 to 6, N was applied with Di-Ammonium Phosphate (DAP) at

planting and from treatment 7 to 12 no N was applied at planting instead TSP (Tri- Supper

Phosphate) was used.”

What about TSP application for treatment 1 to 6? As TSP was applied to all plot, I suggest authors to state “Note that, from treatment 1 to 6, N was applied with Di-Ammonium Phosphate (DAP) at planting and from treatment 7 to 12 no N was applied at planting.” Continue with the rest of sentence seems like no P was applied for treatment 1 to 6 and this change completely the objective of the study. But few lignes after, we can read

“…DAP and TSP were the sources of P2O5 and DAP and urea and DAP were the sources of N…”

There is one more DAP in the sentence.

There is one more “DAP” in the sentence. Rephrase it as :

“…DAP and TSP were the sources of P2O5 and DAP and urea were the sources of N…”

𝐴𝑑𝑗𝑒𝑠𝑡𝑒𝑑 𝑌𝑖𝑒𝑙𝑑, write adjusted yield

“At crop maturity, a subsample from each net plot was harvested at ground level, oven-dried at 70°C until constant weight was reached for dry weight determination and partitioned into straw and grain.”

Why this yield for protein content was not adjusted to 12.5% the moisture?

**Results and Discussion**

**Effect of locations and growing seasons**

From Table 1 to 4, all significance shown is P < 0.01, not P < 0.001

Under Table 3 “**significant at the .01 probability level” one asterisk is smaller than the second. Harmonise

**Yield and yield component of bread wheat**

“Results showed in all locations N split and application time was significantly (*P*<0.01) …” Results shown…”

“Results showed in all locations N split and application time was significantly (*P*<0.01)

affected plant height, total tillers m-2, biomass and grain yield (Table 1).” Add Table 2 also in bracket. Grain and biomass yields are presented in Table 2

“… The possible explanation of the positive effect of split N application compared to single N application at later growth stages is gaseous losses from applied topdressing. …”

This is mainly due to the poor response of plants at advanced growth stages that cause N leaching or volatilization

“Specifically, the highest tiller m-2 at Adet and Debre Elies was obtained from the application of ½ urea at 50% emergence + ½ urea at tillering plus adition of N with DAP at planting, …” Addition instead of adition

“… production of tillers and ears that result in grains [18), …” The citation should be in bracket

Sometimes authors write Wonberema, sometimes Wonberima in tables or Woneberema, what is the right handwritting

Figure 3: The standard deviation/error bars for the protein content are not very visible for 2 and 3 split applications. Change the color. Either put it in red like the curve

“The non-responsiveness of hectoliter weight to N application at Debre Elies was in line with

that of Dawit et al. [53] who reported a non-significant effect of N rates on hectoliter weight of

bread wheat.”

The reference [53] refers to maize not bread. Moreover, the current study focus on the effect of N split and time of application not N rates. So the comparison is not appropriate.

“In all locations N split and time of application significantly affected the grain protein content of wheat (Table 4).”

This sentence should be moved to the beginning of the next section on grain protein content

“[55] ( Iqbal et al., 2012).” Delete the citation in parenthesis

“The highest grain protein content at Woneberema and Debre Elies was absorbed when…” Absorved instead of Obserbed

“… when all N was applied at booting stage (with no application of N at planting) Table 4). This result is parallel to th works of Efretuei et al. [47]” Typing errors in the sentence (parenthesis and “e” miss)

“The absence of a grain protein content reduction as N application was delayed would indicate that either there was sufficient N in the soil to support the plant’s needs during this period or that the plant was able to overcome any temporary deficiency in N that occurred during this period.

Authors (Smith et al., 1991; Mi et al., 2000; Woolfolk et al., 2002), showed that delaying N application till booting, anthesis or flowering increase mainly grain protein content than grain yield

**Correlation between grain yield and other agronomic and quality**

**parameters of wheat**

There are no table 5 and 6 in the manuscript as announced in this section

In table 7, the pearson correlation coefficient is represented by both "r" and "R. I suggest that the authors be consistent and rigorous in writing

**References**

Some references are incomplete. Names of editions and cities/countries for quoted books are missing; numbers and pages of some quoted journals are also missing. Some examples

[1]. FAO (Food and Agriculture Organization). Crop Prospects and Food Situation. 2014; Pp5-7

It is missing the edition and place of publication in this reference

[5]. CSA (Central Statistical Agency) 2018. Agricultural Sample Survey, report on area and

production of major crops. Statistical bulletin……2018 ;. Addis Ababa, Ethiopia

Numero ??? Page?

[14]. Amsal T and Tanner D. Effects of Fertilizer application on N and P uptake, recovery and

use efficiency of bread wheat grown on two soil types in central Ethiopia. Ethiopian

Journal of Natural Resources.2001;

Complete with the number and pages

**MY CONCLUSION ON THE MANUSCRIPT**

Overall, it is a good scientific contribution and provides a solution on N management to wheat farmers especially in Ethiopia. The introduction and materials and methods are sufficiently documented and the conclusion support the study findings. However, the authors lacked rigor in the manuscript writing : many typing errors, grammar and phrasing issues , the journal's instructions to the authors not followed for the citation of figures, the announced tables not included, incomplete and sometimes inappropriate references.

The manuscript can be accepted after correction of identified deficiencies.
