# Supplementary material for: Optimizing time and split application of nitrogen fertilizer to harness grain yield and quality of bread wheat (Triticum Aestivum L.) in northwestern Ethiopia
Source: PLoS One. 2022 Dec 15;17(12):e0279193. doi: 10.1371/journal.pone.0279193 (PMC10045545; doi:10.1371/journal.pone.0279193)
Supplement: Attachment — Submitted filename: Response to Reviewers.docx [file pone.0279193.s004.docx]

Response to reviewer’s ad editors

| Reviewer comment | Author response |
| --- | --- |
| ***Reviewer 1*** | |
| Optimizing Nitrogen Fertilizer Split and Time of Application to Bread Wheat (Triticum Aestivum L.) Grain Yield and Quality in Northwestern Ethiopia  PLOS ONE | ***Optimizing Time and Split Application of Nitrogen Fertilizer to harness Grain Yield and Quality of Bread Wheat*** *(Triticum Aestivum* L.*)* ***in Northwestern Ethiopia*** |
| Sowing is appropriate word | ***Accepted the comment*** |
| How you can know, there was no any genotype tria in the experiment | ***The yield and quality are varied across the environments*** |
| Consistency, once planting and the other time sowing. Anyways sowing is the appropriate word | ***corrected*** |
| Is it the amount of nitrogen fertilizer applied in split increased you mean ?? If it is so please add words indicating amount/rates of N application in split form? | At all locations, grain protein content decreased as the number of N split application increased one to 3 times. |
| What about the Economic analaysis?? Just include it as it is very crucial to recommend for farmers based on the cost they incur and benefits gained?? | included |
| Key words should be in alphabetic | **Key words:** grain protein content; grain yield; hectoliter weight; wheat |
| is it the right reasoning? Instead it is due to high nitrogen fertilizer use efficiency which increases the canopy/leaf area and it again increases the photosynthesis rate/photoassimilate that increases the grain weight. | Corrected |
| Reference | Corrected |
| Reviewer 2 | |
| It’s missing full stop and the end of the first sentence. |  |
| Name the treatments | Descried I the document |
| What is the difference between all urea at booting and all N at booting | **Same** but to differentiate treatment 1 to 6, (N was applied with Di-Ammonium Phosphate (DAP) at sowing) and treatment 7 to 12 (no N was applied at sowing instead TSP (Tri- Supper Phosphate) was used). Please see in the materials and methods |
| it can concluded, correct sentence is “It can be concluded”. | **Corrected based on the comments** |
| “Despite its importance, the productivity of wheat in Ethiopia under rain fed season is low  particularly in Amhara region (2.79)” what is the unit of 2.79? | 2.79 tone/ha |
| “Low soil fertility, improper fertilizer application, lack of appropriate seeding, feretilizer  rate; and planting methods and pests are among the major constraints limiting wheat production  in Ethiopia [12, 13].” | Low soil fertility, improper fertilizer application, lack of appropriate seeding rate, fertilizer rate, planting methods and pests are among the major constraints limiting wheat production in Ethiopia [12, 13]. |
| To reduce these  challenges increasing the N use efficiency through different strategies such as (i) use of  appropriate type of fertilizer and application time during the crop growing period, (ii) application  of optimum N fertilizer rate, (iii) appropriate method of application, (iv) use of appropriate  cultivar and (v) application at the appropriate climatic conditions [21].” | To reduce these challenges increasing the N use efficiency through different strategies is necessary. Some of the strategies includes |
| “Thus, the main objective of this research was to determine the  appropriate N fertilizer application time for maximum bread wheat yield production with  optimum protein contenet.” Typing error prtein contenet. Write protein content… | Thus, the main objective of this research was to determine the appropriate N fertilizer application time for maximum bread wheat yield production with optimum protein content. |
| “…wheat growing stags with various ratios of N across…” write growing stages | corrected |
| A field experiment was conducted in three wheat production potentail areas of…  Potential areas of… | A field experiment was conducted in three wheat production potential areas |
| **“…** districts of Amhara Region, Ethiopia (Figure 1) …” Follow the journal's instructions for citing figures and tables in the manuscript. |  |
| “Generally, the rainfall in the study areas follows a dominantly unimodal distribution with the  main peak in June to September,” Does this mean June is rainy as July, August and September? Main peak should be the rainiest month | No sir, highest rainfall has been found in those months |
| The mean annual rainfall mentioned here is less than the rainfall recorded during the growing season in each of the 3 areas (Figure 2). How do authors explain this? Are they mean annual rainfall calculated over a period? | Yes sir, most of the time The mean annual rainfall over the period (30 years) is less than the rainfall recorded during the growing season in each of the 3 areas. |
| “In Debre Elias and Wonberema, the rain falls increased from 2014 and 2015 by  71.43% and 14.29%, respectively, ...” write ...from 2014 to 2015 by 71.43% and 14.29%, respectively, | In Debre Elias and Wonberema, the rain fall increased from 2014 to 2015 by 71.43% and 14.29%, respectively, while at Adet it was relatively constant across years |
| At Adet the soil textural class is clay with the soil pH, OC (g kg-1), TN (mg kg-1), Ava.P (mg kg-1), CEC (meq100g-1) of 5.4, 10.7, 966, 27.69 and 31.2, respectively [35]. The soil pH , OC(%), TN (%), Ava.P (ppm) and Av.K (ppm) at Wonberema were 5.7, 3.1, 0.3, 369 and 127, respectively with sandy loam textural lass [36]. The soil pH, OM (%), TN (%), Ava.P (mg kg-1) and CEC (Cmole(+) kg-1) at Debre Elias were 5.12, 2.63, 0.14, 2.53 and 25.02, respectively”  Better to present this in a table and make comments.  Having 966 mgN/kg of soil at Adet, no need to conduct such study on N splitting there. The soil contents much N than plant required. Chek this result. Same remark for Ava.P at Wonderema (369mg/kg). Why authors present TN results in different unit (mg kg-1 and %) in the same study. Please harmonize it. ppm = mg kg-  Morever, the soil characterics presented are different from location to location. For instance, at Wonberema, authors don’t present the soil texture and CEC value and at Debre no soil texture presented. Harmonize this since soil texture and CEC impact plant response | At Adet the soil textural class is clay [35] while at Wonberema [36] and Debre Elias [37] the soil textural class is sandy loam. The experimental site at Adet constitutes pH 5.4, organic carbon 2.47% Total nitrogen 0.8%, available phosphorus 1.98 ppm and cation exchange capacity 31.2 Cmole(+) kg^-1^ [35] while the respective soil nutrients at Debre Elias were 5.12, 2.63%, 0.14% , 2.53pp and 25.02 Cmole(+) kg^-1^ [37]. At Wonberema, pH, organic carbon, total nitrogen and available phosphorus were 572, 3.1%, O.3%, 3.69ppm, respectively [36]. |
| I suggest authors to present this in table to facilitate the reading to readers For instance, a table like the following Treatment \| Nitrogen application \| \|; \| 1^st^ application \| 2^nd^ application \| 3^rd^ application; 1 \| \| \|; 2 \| \| \| Note that, from treatment 1 to 6, P was applied in the form of Di-Ammonium Phosphate (DAP) while from treatment 7 to 12, P was applied in the form of TSP (Tri- Supper Phosphate) at sowing. Thus, N was applied at sowing only in the former treatments (1 to 6) “Note that, from treatment 1 to 6, N wa++ s applied with Di-Ammonium Phosphate (DAP) at planting and from treatment 7 to 12 no N was applied at planting instead TSP (Tri- Supper Phosphate) was used.” “Note that, from treatment 1 to 6, N was applied with Di-Ammonium Phosphate (DAP) at planting and from treatment 7 to 12 no N was applied at planting instead TSP (Tri- Supper Phosphate) was used.” What about TSP application for treatment 1 to 6? As TSP was applied to all plot, I suggest authors to state “Note that, from treatment 1 to 6, N was applied with Di-Ammonium Phosphate (DAP) at planting and from treatment 7 to 12 no N was applied at planting.” Continue with the rest of sentence seems like no P was applied for treatment 1 to 6 and this change completely the objective of the study. But few lignes after, we can read “…DAP and TSP were the sources of P2O5 and DAP and urea and DAP were the sources of N…” There is one more DAP in the sentence. There is one more “DAP” in the sentence. Rephrase it as : “…DAP and TSP were the sources of P2O5 and DAP and urea were the sources of N…” | Table 2: Description of the experimental treatments **TrtNo.** \| **Nitrogen application** \| \|; \| **1^st^ application** \| **2^nd^ application** \| **3^rd^ application**; **1** \| 1/3^rd^ urea at 50% emergence \| ½ urea at tillering \| -; **2** \| ½ urea at tillering \| ½ urea at booting \| -; **3** \| 1/3^rd^ urea at 50% emergence \| 1/3^rd^ urea at tillering \| 1/3^rd^ urea at booting; **4** \| 2/3^rd^ urea at tillering \| 1/3^rd^ urea at booting \| -; **5** \| All urea at tillering \| - \| -; **6** \| All urea at booting \| - \| -; **7** \| All N at tillering \| - \| -; **8** \| All N at booting \| - \| -; **9** \| ½ N at sowing \| ½ N at tillering \| -; **10** \| ½ N 50% emergence \| ½ N at tillering \| -; **11** \| 1/3^rd^ N at 50% \| 1/3^rd^ N at tillering \| 1/3^rd^ N at booting; **12** \| ½ N at tillering \| ½ N at booting \| - TrtNo.−Treatment number. Sowing−Zadoks Growth Stage 00; emergence−Zadoks Growth Stage 11-18; tillering−-Zadoks Growth Stage 21-26); booting−Zadoks Growth stage 41- 45. Note that, Di-Ammonium Phosphate (DAP) and TSP (Tri- Supper Phosphate) were the sources of P2O5 and DAP and UREA were the sources of N. However, at sowing P_2_O_5_ was applied in the form of DAP from treatment 1 to 6, while from treatment 7 to 12, P_2_O_5_ was applied in the form of TSP. |
| “At crop maturity, a subsample from each net plot was harvested at ground level, oven-dried at 70°C until constant weight was reached for dry weight determination and partitioned into straw and grain.”  Why this yield for protein content was not adjusted to 12.5% the moisture? | From the above dried, cleaned and adjusted yield a subsample from each net plot was taken and milled. Then grain N content of the samples were determined using the micro-Kjeldahl method as stated by American Association of Cereal Chemists (AACC) [41]. The protein content of wheat flour samples for each treatment was performed by following AACC [42] using a 5.7 conversion factor as follows and expressed in dry matter %. |
| From Table 1 to 4, all significance shown is P < 0.01, not P < 0.001  Under Table 3 “**significant at the .01 probability level” one asterisk is smaller than the second. Harmonise | Corrected based on the comments |
| “Results showed in all locations N split and application time was significantly (*P*<0.01) …” Results shown…”  “Results showed in all locations N split and application time was significantly (*P*<0.01)  affected plant height, total tillers m-2, biomass and grain yield (Table 1).” Add Table 2 also in bracket. Grain and biomass yields are presented in Table 2  “… The possible explanation of the positive effect of split N application compared to single N application at later growth stages is gaseous losses from applied topdressing. …” | Results shown in all locations N split and application time was significantly (*P*<0.01) affected plant height, total tillers m^-2^, biomass and grain yield (Table 3 and 4).  The possible explanation of the positive effect of split N application compared to single N application at later growth stages is gaseous losses from applied topdressing |
| This is mainly due to the poor response of plants at advanced growth stages that cause N leaching or volatilization  “Specifically, the highest tiller m-2 at Adet and Debre Elies was obtained from the application of ½ urea at 50% emergence + ½ urea at tillering plus adition of N with DAP at planting, …” Addition instead of adition | This is mainly due to the poor response of plants at advanced growth stages that cause N leaching or volatilization. Specifically, the highest tiller m^-2^ at Adet and Debre Elies was obtained from the application of ½ urea at 50% emergence + ½ urea at tillering plus addition of N with DAP at planting, |
| “… production of tillers and ears that result in grains [18), …” The citation should be in bracket  Sometimes authors write Wonberema, sometimes Wonberima in tables or Woneberema, what is the right handwritting | [18]  Wonberema |
| Figure 3: The standard deviation/error bars for the protein content are not very visible for 2 and 3 split applications. Change the color. Either put it in red like the curve | Corrected |
| “The non-responsiveness of hectoliter weight to N application at Debre Elies was in line with  that of Dawit et al. [53] who reported a non-significant effect of N rates on hectoliter weight of  bread wheat.” | Corrected |
| “In all locations N split and time of application significantly affected the grain protein content of wheat (Table 4).”  This sentence should be moved to the beginning of the next section on grain protein content | Corrected |
| “[55] ( Iqbal et al., 2012).” Delete the citation in parenthesis  “The highest grain protein content at Woneberema and Debre Elies was absorbed when…” Absorved instead of Obserbed | Corrected  The highest grain protein content at Wonberema and Debre Elies was obtained when ½ urea was applied at each wheat tillerng and booting stages |
| **Correlation between grain yield and other agronomic and quality**  **parameters of wheat**  There are no table 5 and 6 in the manuscript as announced in this section  In table 7, the pearson correlation coefficient is represented by both "r" and "R. I suggest that the authors be consistent and rigorous in writing | Corrected based on the comments |
| **References**  Some references are incomplete. Names of editions and cities/countries for quoted books are missing; numbers and pages of some quoted journals are also missing. Some examples  [1]. FAO (Food and Agriculture Organization). Crop Prospects and Food Situation. 2014; Pp5-7  It is missing the edition and place of publication in this reference  [5]. CSA (Central Statistical Agency) 2018. Agricultural Sample Survey, report on area and  production of major crops. Statistical bulletin……2018 ;. Addis Ababa, Ethiopia  Numero ??? Page?  [14]. Amsal T and Tanner D. Effects of Fertilizer application on N and P uptake, recovery and  use efficiency of bread wheat grown on two soil types in central Ethiopia. Ethiopian  Journal of Natural Resources.2001;  Complete with the number and pages | All references are Corrected |
