# Supplementary material for: Optimizing time and split application of nitrogen fertilizer to harness grain yield and quality of bread wheat (Triticum Aestivum L.) in northwestern Ethiopia
Source: PLoS One. 2022 Dec 15;17(12):e0279193. doi: 10.1371/journal.pone.0279193 (PMC10045545; doi:10.1371/journal.pone.0279193)
Supplement: Attachment — Submitted filename: Comment_Plos_one second review.docx [file pone.0279193.s005.docx]

some minor corrections are still necessary. Below are these corrections

The mean annual rainfall mentioned here is less than the rainfall recorded during the growing season in each of the 3 areas (Figure 2). How do authors explain this? Are they mean annual rainfall calculated over a period? Yes sir, most of the time. The mean annual rainfall over the period (30 years) is less than the rainfall recorded during the growing season in each of the 3 areas.

For better understanding indicate the period over which the mean annual rainfall is calculated

… while the respective soil nutrients at Debre Elias were 5.12, 2.63%, 0.14% , 2.53pp and 25.02 Cmole(+) kg-1 [37]. At Wonberema, pH, organic carbon, total nitrogen and available phosphorus were 572, 3.1%, O.3%, 3.69ppm, respectively [36].

Still have typing errors in this sentence. Correct them

2.53 ppm

pH at Wonberema was 5.72

Total nitrogen at Wonberema 0.3% not O.3% (the zero written with letter “O”

**Data collection**

Ajusted yield instead of adujested yield

**Results and discussion**

This is mainly due to the poor response of plants at advanced growth stages that cause N leaching or volatilization. This sentence supports the following statement “The possible explanation of the positive effect of split N application compared to single N application at later growth stages is gaseous losses from applied topdressing. It is not to explain the increase in the number of tillers with split applications. Please remove it

In table 6, the pearson correlation coefficient ‘r’ is written in the same table with upper case R and lower case r. Be coherent and rigourous. Either “r” or “R” not both at the same time
