# Supplementary material for: Optimizing time and split application of nitrogen fertilizer to harness grain yield and quality of bread wheat (Triticum Aestivum L.) in northwestern Ethiopia
Source: PLoS One. 2022 Dec 15;17(12):e0279193. doi: 10.1371/journal.pone.0279193 (PMC10045545; doi:10.1371/journal.pone.0279193)
Supplement: Attachment — Submitted filename: Response to reviewers.docx [file pone.0279193.s006.docx]

**Response to reviewer**

The mean annual rainfall mentioned here is less than the rainfall recorded during the growing season in each of the 3 areas (Figure 2). How do authors explain this? Are they mean annual rainfall calculated over a period? Yes sir, most of the time. The mean annual rainfall over the period (30 years) is less than the rainfall recorded during the growing season in each of the 3 areas.

For better understanding indicate the period over which the mean annual rainfall is calculated

**Response to Reviewers:** It has a long year mean (30 years) annual rainfall of 1211mm with a minimum and maximum temperature of 11.57^°^C and 26.89^°^C, respectively

… while the respective soil nutrients at Debre Elias were 5.12, 2.63%, 0.14% , 2.53pp and 25.02 Cmole (+) kg-1 [37]. At Wonberema, pH, organic carbon, total nitrogen and available phosphorus were 572, 3.1%, O.3%, 3.69ppm, respectively [36].

Still have typing errors in this sentence. Correct them

2.53 ppm

pH at Wonberema was 5.72

Total nitrogen at Wonberema 0.3% not O.3% (the zero written with letter “O”

**Response to Reviewers**. At Adet the soil textural class is clay [35] while at Wonberema [36] and Debre Elias [37] the soil textural class is sandy loam. The experimental site at Adet constitutes an average pH of 5.4, organic carbon of 2.47%, total nitrogen of 0.8%, available phosphorus of 1.98 ppm and cation exchange capacity of 31.2 Cmole (+) kg^-1^ [35] while the respective soil nutrients at Debre Elias were 5.12, 2.63%, 0.14%, 2.53ppm and 25.02 Cmole(+) kg^-1^ [37]. At Wonberema, pH, organic carbon, total nitrogen and available phosphorus were 5.72, 3.1%, 0.3%, 3.69ppm, respectively [36].

**Data collection**

Ajusted yield instead of adujested yield

**Response to Reviewers**. Corrected based on the comment

**Results and discussion**

This is mainly due to the poor response of plants at advanced growth stages that cause N leaching or volatilization. This sentence supports the following statement “The possible explanation of the positive effect of split N application compared to single N application at later growth stages is gaseous losses from applied topdressing. It is not to explain the increase in the number of tillers with split applications. Please remove it

**Response to Reviewers**. Corrected based on the comment

In table 6, the pearson correlation coefficient ‘r’ is written in the same table with upper case R and lower case r. Be coherent and rigourous. Either “r” or “R” not both at the same time

**Response to Reviewers**. Corrected based on the comment
